# Qualitative Accounts of School-Aged Children’s Diets during the COVID-19 Pandemic in Rural, Central, Kenya

**DOI:** 10.3390/nu13103543

**Published:** 2021-10-09

**Authors:** Megan Jarman, Henriette Zeidler, Laura Shapiro, Rebecca Clarke, Haatembo Mooya, Barnabas Simatende, Danielle Matthews, Grace Koteng, Pamela Wadende, Claire Farrow

**Affiliations:** 1 School of Psychology, College of Health and Life Sciences, Aston University, Birmingham B4 7ET, UK; m.jarman@aston.ac.uk (M.J.); hzeidler@gmail.com (H.Z.); L.R.Shapiro@aston.ac.uk (L.S.); clarker3@aston.ac.uk (R.C.); 2 Department of Psychology, Humanities and Social Sciences, University of Zambia, Lusaka 32379, Zambia; haatembo.mooya@unza.zm (H.M.); lifeisgoodregardless@gmail.com (B.S.); 3 Department of Psychology, University of Sheffield, Sheffield S10 2TN, UK; Danielle.matthews@sheffield.ac.uk; 4 Department of Educational Psychology, School of Education and Human Resource Development, Kisii University, Kericho 20200, Kenya; g.koteng@gmail.com

**Keywords:** qualitative, nutrient intakes, COVID-19, rural, Kenya, children

## Abstract

The COVID-19 pandemic has caused disruption to food security in many countries, including Kenya. However, the impact of this on food provision to children at an individual level is unknown. This small study aimed to provide a qualitative snapshot of the diets of children during the COVID-19 pandemic. During completion of 24-h food recalls, with 15 families with children aged 5–8 years, caregivers were asked about changes they had made to foods given to their children due to the pandemic. Food recalls were analysed to assess nutrient intakes. Qualitative comments were thematically analysed. Most of the families reported making some changes to foods they provided to their children due to COVID-19. Reasons for these changes fell into three themes, inability to access foods (both due to formal restriction of movements and fear of leaving the house), poorer availability of foods, and financial constraints (both decreases in income and increases in food prices). The COVID-19 pandemic has affected some foods parents in rural Kenya can provide to their children.

## 1. Introduction

Access and availability to affordable nutritious foods has often been a challenge in rural Kenya, as highlighted by studies reporting that the prevalence of food insecurity is as high as 63–76% [1,2]. In March 2020, the COVID-19 global pandemic reached Kenya, and in response, the government, in line with guidance from the World Health Organisation, implemented restrictions to reduce the spread of the virus. Such regulations included boarder closures, social distancing, movement restrictions and closure of non-essential services [3]. These restrictions are likely to have had unintended effects on food access and availability via the disruption of food systems, and reduced incomes of families who are no longer able to find work [4]

Whilst rapid analyses have been conducted to estimate the financial effects on families [3], there has been little consideration of food availability and access issues, and impact on the dietary intakes of children in rural Kenya at the individual level. As part of a project already taking place with families in rural, central Kenya, we were able to collect qualitative data from families to provide a snapshot of some issues parents faced when feeding their children during the COVID-19 pandemic. In this short report, we aim to describe the challenges these families faced and changes they made to foods they fed their children at this time. 

## 2. Materials and Methods

### 2.1. Participants

Participants included 15 families of children aged between 4.8 and 7.6 years. Families were recruited from two rural communities in Laikipia East in central Kenya. Both communities (Chuma and Matanya) are located about 15 km south-west of Nanyuki, the nearest town. Participants were recruited as part of a larger ongoing study funded by the UKRI Global Challenges Research Fund (via ESRC, see funding sources), with 80 families taking part in multiple mealtime observations at home and school in Kenya and Zambia.

### 2.2. Procedure

Researchers used existing contacts to local primary schools, whose preschool teachers verbally invited eligible families to take part. Where families were interested in the study, they were given more details about the procedures by local researchers who shared information sheets and consent forms with participants, reading out and explaining items whenever necessary. The study team have a long-standing relationship with these communities which aided participation. Participating parents gave written informed consent, either by signing or providing a thumb print, depending on literacy levels. For the measures reported in this study, parents provided details in interviews, prior to local COVID-19 lockdown restrictions, or over the telephone after lockdown restrictions. Child anthropometric data were collected in schools by trained teachers and research assistants after the lockdown restrictions were lifted (March 2021).

#### 2.2.1. Qualitative Statements

Parents were asked open-ended questions about the impact of the pandemic on the child’s food provision. In particular, as part of each 24-h recall, parents were asked whether the child’s food was typical on that day (and if not, what was different and why), and for each food/drink item whether there was any specific impact of COVID-19 on what the child had consumed (i.e., on the food which the family was able to provide). Data were entered into NViVo version 12 [5] for analysis.

#### 2.2.2. Demographics

The Kenyan Demographics and Health Survey Tool (https://dhsprogram.com/ accessed on 1 June 2020), education and poverty indicators were adapted and used to gain information about maternal and paternal education and family demographics. 

#### 2.2.3. Dietary Recall Data

The dietary recall procedure was adapted to local standards in line with recommendations from the GloboDiet-Africa team [6] and extensively piloted with a team of local preschool teachers and research assistants. Parents completed the 24 h recalls on behalf of their child. Parents completed a 24-h dietary recall over the telephone with a local, trained, researcher. Recalls were completed on three separate days (two weekdays, and one Sunday) in the course of two weeks. Three-repeated 24-h recalls has been shown to yield similar nutrient intakes as three days of prospective weighed food diaries in children in rural Kenya [7], furthermore a review of use of 24 h recalls in low-income countries shows that fewer days of reporting is needed due to the limited variability in individual dietary intakes [8]. Parents provided information about all foods that their child had consumed in the 24-h period preceding the interview. In addition to the information concerning the time and location of snacks and/or meals, the foods consumed (including foods, beverages, condiments, sauces and spreads), brand information, preparation methods, and portion sizes were collected. Grams/day of each food item was calculated for each child and the Food Composition Tables from Kenya [9] were then used to calculate mean daily nutrient intakes. These were compared to nutrient intake recommendations as outlined in the joint FAO/WHO 2002 report [10] and each child was identified as having an adequate or inadequate intake for their age category. The joint FAO/WHO 2004 report on energy requirements [11], was used to determine those with adequate or inadequate energy (kcal) intakes, based on age, sex and weight. 

#### 2.2.4. Anthropometrics

Child height and weight were collected in schools by a team of teachers/research assistants trained in using measuring materials. The scales used were Ramtons RM304, which were re-calibrated before each measurement. Height was measured using a yardstick after marking the child’s height on a wall. All measures were taken by one team member and confirmed by a second member of the team. Children were weighed in their school uniforms with shoes removed. Child height and weight were converted to weight for height for age Z scores (WHZ), and height for age (HFA) Z scores, using the WHO AnthroPlus software version 1.0.4 [12]. Children were classed as underweight if their WHZ z scores were ≤−2SD. Having overweight or obesity were defined as >2SD and >3SD, respectively. Stunting was defined as HFA ≤ −2SD.

### 2.3. Analysis 

The qualitative data were collected by local, trained fieldworkers in the language requested by the participant (either Kikuyu, Kiswahili, or English). The fieldworkers were fluent in all three languages, and they translated the data from the local language into English. The qualitative data were then analysed by two UK researchers (RC and MJ) through a process of data familiarisation, independent and inductive coding, and grouping of codes into themes. Codes and themes emerging from the data were discussed with researchers in Kenya (PW and HZ) to ensure that results were not biased by cultural assumptions. Consensus on coding and theme generation was reached through discussion. Reports of particular food/drink items that were altered in the diet were extracted from the statements to produce a list of commonly excluded foods. The summary statistics of the quantitative data (*n*, percentages, mean (sd)) were analysed in Stata version 14.0 [13].

## 3. Results

### 3.1. Quantitative Analyses

Participants were 15 children: seven girls and eight boys. Fourteen of the children had siblings. Eight children were recruited from Chuma village and seven from the Matanya village. The main caregiver of 12 of the children was the mother, for two it was the father and for one the great-grandmother. Children’s mothers reported an average of 9.9 (SD 2.5) years in school and fathers reported an average of 9.8 (SD 2.4) years (all numbers include two years of preschool). Thirteen children lived in timber houses, one stone and one iron-sheet housing. Six children had access to electricity at home (three had solar panels and three had Kenya Power supplied energy). Only one child’s home had a private water source, and for the others the average walking distance to the water supply was 27 minutes walking (SD 14).

One child presented as underweight, one overweight and one with obesity. All other children (*n* = 12) fell within the healthy WHZ category. Furthermore, no children in this cohort were classified as wasted or stunted.

Median nutrient intakes, and the number of children with inadequate intakes over the three days of reporting are shown in Table 1. Ten of the 15 children had inadequate calcium intakes and nine children had inadequate niacin intakes. Whilst some children had inadequate intakes of the other nutrients, fewer than half of the sample were inadequate in this regard.

### 3.2. Qualitative Analyses

In total, 11 of the 15 participants discussed making changes to foods usually provided to their child due to the COVID-19 pandemic. The most commonly reduced or excluded food items were fruits, tomatoes, meat and sugar. Qualitative analysis generated three core themes which reflect why changes were made due to the COVID-19 pandemic: (1) barriers to access, (2) financial changes and (3) limited availability.

#### 3.2.1. Barriers to Access

Parents revealed that accessing food was difficult as they were either afraid to travel to the market in case of catching the virus, or the restriction of movement meant that visiting the market was not allowed:

“*Yes, we were unable to get some ingredients because of the fear to get to the market due to covid-19*”

(Participant 7)

“*We would have wished to have maybe fruits in their meal but it has become hard to get to the market due to the fear of being infected*”

(Participant 8)

“*We used to buy fruits for the child but this has become hard since movements to the market have been minimized*”

(Participant 16)

#### 3.2.2. Financial Changes

Changes to families’ financial circumstances caused by a reduction in family income due to job loss, and an increase in the cost of the foods at the market, led to alterations in diet:

“*In times like this when we receive a visitor, we could add some meat to the stew but it has become hard to get some wage and casual works due to the effect of the virus*”

(Participant 15)

“*Yes, the amount of milk used to prepare the tea has reduced since the virus broke. This is because we cannot afford to buy more because jobs have become scarce and we rely on wage work*”

(Participant 6)

“*When cooking meat stew we usually include tomatoes but we did not because they have turned to be expensive*”

(Participant 9)

#### 3.2.3. Limited Availability

Participants identified that travel restrictions caused by the COVID-19 pandemic limited the availability of some foods:

“*Yes, we were not able to add tomatoes to the food as the pandemic has made it hard for the vendors to transport them together with other commodities… because of various restrictions*”

(Participant 6)

“*We are forced to take whatever is available as the pandemic has changed life*”

(Participant 2)

**Figure 1:**
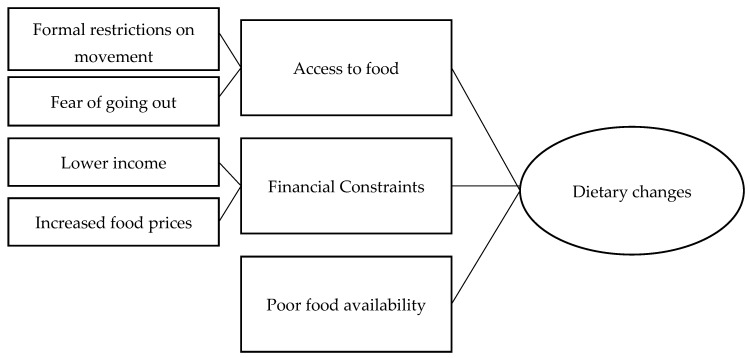
Thematic map showing the main themes and subthemes of reasons for making dietary changes.

The summary of the themes is shown in the thematic map (Figure 1).

## 4. Discussion

In this short report, we aimed to present a snapshot of the food provision to some school-aged children in rural, central, Kenya during the COVID-19 pandemic. We found that most of the families we spoke to had to make changes to the food they provided their children, either due to reduced household budgets, and/or a rise in the cost of some foods, poorer access to the market places, and/or poorer availability of foods. Furthermore, whilst this small cohort were mostly of a healthy weight for their height and age, there were some important micronutrients lacking from the diets of these children over the same time-period.

In the FAO policy briefing describing the impact of COVID-19 on the food and nutrition security of families in Kenya, the availability and access to foods was discussed [4]. At a national level it was reported that there was no impact on food availability with “supermarkets and food stores not running out of stock” [4]. Whilst there was mention of a possible impact on market availability, this was focused on those in an urban or peri-urban setting. However, at a rural level, some of the families in our study had noticed a reduction in the availability of some foods, citing the ban on movement as a potential reason for food vendors having limited stock. In line with the policy briefing however, was the discussion of limited food access and financial constraints, with food prices rising in conjunction with income losses. The informal work sector accounts for 83.7% of employment in Kenya [5], and in our study sample two thirds of families reported relying on ‘wage work’ or ‘selling local produce’. A recent study focusing on the financial impact of COVID-19 on food security in Kenyan families also documented the double-edged sword of increasing food cost with decreasing incomes [5]. Seventy-three respondents reported lower regular income due to COVID-19 and 40% reported changing their dietary patterns as a result, although which foods were reduced/eliminated was not reported; they employed the use of social media to collect data, and therefore had a more affluent sample with internet access, and were mainly from urban areas. Our study has also suggested that limited food access was not only due to financial constraints and formal restrictions on movement, but participants also reported fear of becoming infected with COVID-19 as a reason for limiting travel to the marketplace. A reduction in going out due to fear of the pandemic was highlighted in an article considering impacts on physical activity [14], but to the best of our knowledge the effect of fear on limiting food acquisition has not previously been considered in the literature. In a recent (2020) debate piece, highlighting potential impacts of COVID-19 on dietary and physical behaviours related to non-communicable diseases in Urbanising countries such as Kenya, the authors state that the measures employed to curb the spread of COVID-19 would likely have a multipronged effect on diets through impacting family incomes, food availability, price, and access [15]. Although the Kenyan government aimed to limit food insecurity by auditing the supply and prices of food staples and prioritising vulnerable families, our empirical data showed that food access and availability were still impacted for some rural families.

Our participants reported that fruits, tomatoes, meats and sugar were among the foods most commonly reduced or removed from the diet due to the COVID-19 pandemic. More perishable foods, such as fruits and tomatoes, may have been more difficult for market vendors to acquire, thus limiting their availability and increasing their price. In contrast, although meats and sugar were still readily available, these may be considered more expendable when families food budgets are reduced. Over half the participants had inadequate niacin intakes, which may partly reflect the lack of meat intake in most of their diets. Inadequate calcium intakes were also noted, which could be a result of low dairy intakes; whilst every child consumed a little milk every day in tea, they did not have other forms of dairy in their diet (data not shown). 

The sample size is a clear limitation of our study, although qualitative studies do not intend to be representative of whole populations. Instead, we aimed to provide qualitative insights into school-aged children’s diets during the COVID-19 pandemic in two villages in rural Kenya. It is a strength that we were able to collect data with rural families, which was due to the long-standing, trusting, relationship which had already been forged between our study team and the communities. These communities tend to be underserved in studies.

## 5. Conclusions

The COVID-19 pandemic may have adversely effected foods that were provided to children in some rural locations in Kenya. Barriers to food provision caused by the pandemic included reduced access to marketplaces, both due to fear of infection and formal movement restrictions, lower family incomes, increasing food prices, and poor availability of produce on offer. These provide some insight into some of the immediate implications of the pandemic, highlighting which types of foods tend to be reduced when hardship occurs. This may be taken into consideration in future studies of food-related relief procedures during crises, in particular how such events may exacerbate food availability and access in rural communities. 

## Figures and Tables

**Table 1:** Description of nutrient intakes over three days of reporting during the COVID-19 pandemic.

Nutrient	Median (Range)	N Inadequate Intake
Kcal	1613.7 (868.1–2638.2)	6
Calcium (mg)	506.7 (266.1–1090.4)	10
Iron (mg)	11.2 (5.7–30.4)	1
Magnesium (mg)	274.0 (128.8–420.6)	0
Zinc (mg)	6.9 (4.2–9.9)	2
Selenium (mcg)	24.9 (9.9–35.6)	0
Vitamin A Retinol Equivalents (mcg)	246.1 (134.6–464.7)	6
Thiamin (mg)	0.7 (0.3–2.3)	5
Riboflavin (mg)	1.3 (0.6–2.1)	2
Niacin (mg)	7.8 (4.3–17.9)	9
Folate (mcg)	331.4 (171.2–573.7)	3
Vitamin B12 (mcg)	2.1 (0.9–5.7)	2
Vitamin C (mg)	55.1 (17.1–105.2)	5

## Data Availability

The data that support the findings of this study are openly available in the UK Data Archive at http://reshare.ukdataservice.ac.uk/855241/ accessed on 10 September 2021.

## References

[1] Shinsugi C., Matsumura M., Karama M., Tanaka J., Changoma M., Kaneko S. (2015). Factors associated with stunting among children according to the level of food insecurity in the household: A cross-sectional study in a rural community of Southeastern Kenya. BMC Public Health.

[2] Nagata J.M., Fiorella K.J., Salmen C.R., Hickey M.D., Mattah B., Magerenge R., Milner E.M., Weiser S.D., Bukusi E.A., Cohen C.R. (2015). Around the Table: Food Insecurity, Socioeconomic Status, and Instrumental Social Support among Women Living in a Rural Kenyan Island Community. Ecol. Food Nutrition..

[3] Kansiime M.K., Tambo J.A., Mugambi I., Bundi M., Kara A., Owuor C. (2021). COVID-19 implications on household income and food security in Kenya and Uganda: Findings from a rapid assessment. World Dev..

[4] Demeke M., Kariuki J., Wanjiru M. Assessing the Impact of COVID-19 on Food and Nutrition Security and Adequacy of Responses in Kenya. FAO Policy Briefing May 2020. https://evidencefrontiers.com/wp-content/uploads/2020/05/Policy-Brief_Assessing-the-Impact-of-COVID_19-on-Food-and-Nutrition-Security-1.pdf.

[5] (2018). QSR International Pty Ltd. NVivo (Version 12). https://www.qsrinternational.com/nvivo-qualitative-data-analysis-software/home.

[6] Aglago E.K., Landais E., Nicolas G., Margetts B., Leclercq C., Allemand P., Aderibigbe O., Agueh V.D., Amuna P., Annor G.A. (2017). Evaluation of the international standardized 24-h dietary recall methodology (GloboDiet) for potential application in research and surveillance within African settings. Glob. Health.

[7] Kigutha H.N. (1997). Assessment of dietary intake in rural communities in Africa: Experiences in Kenya. Am. J. Clin. Nutr..

[8] Gibson R.S., Charrondiere U.R., Bell W. (2017). Measurement errors in dietary assessment using self-reported 24-hour recalls in low-income countries and strategies for their prevention. Adv. Nutr..

[9] FAO/Government of Kenya Kenya Food Composition Tables. Nairobi. 2018. 254. http://www.fao.org/3/I9120EN/i9120en.pdf.

[10] World Health Organisation (2004). Food and Agriculture Organisation of the United Nations. Vitamin and Mineral Requirements in Human Nutrition.

[11] United Nations University, World Health Organization (2004). Food and Agriculture Organization of the United Nations. Human Energy Requirements: Report of a Joint FAO/WHO/UNU Expert Consultation.

[12] (2007). World Health Organistion AnthroPlus Software. Version 1.0.4. https://www.who.int/tools/growth-reference-data-for-5to19-years/application-tools.

[13] (2015). Stata Statistical Software. Release version 14.0; StataCorp, College Station, Texas USA. https://www.stata.com/stata14/.

[14] Ben Hassen T., El Bilali H., Allahyari M.S. (2020). Impact of COVID-19 on Food Behavior and Consumption in Qatar. Sustainability.

[15] Oni T., Micklesfield L.K., Wadende P., Obonyo C.O., Woodcock J., Mogo E.R., Odunitan-Wayas F.A., Assah F., Tatah L., Foley L. (2020). Implications of COVID-19 control measures for diet and physical activity, and lessons for addressing other pandemics facing rapidly urbanising countries. Glob. Health Action.

